# On the flexible needle insertion into the human liver

**DOI:** 10.1038/s41598-021-89479-8

**Published:** 2021-05-13

**Authors:** Veturia Chiroiu, Nicoleta Nedelcu, Doina Pisla, Ligia Munteanu, Cristian Rugină

**Affiliations:** 1 Institute of the Solid Mechanics, Romanian Academy, Bucharest, Romania; 2 Technical University of Cluj-Napoca, Cluj-Napoca, Romania

**Keywords:** Mechanical engineering, Mathematics and computing

## Abstract

In the present research, the navigation of a flexible needle into the human liver in the context of the robotic-assisted intraoperative treatment of the liver tumors, is reported. Cosserat (micropolar) elasticity is applied to describe the interaction between the needle and the human liver. The theory incorporates the local rotation of points and the couple stress (a torque per unit area) as well as the force stress (force per unit area) representing the chiral features of the human liver. To predict the deformation of the needle and the liver, the elastic properties of the human liver have been evaluated. Outcomes reveal that considering smaller deformations of the needle and the liver results in better needle navigation mechanism. The needle geometry can enhance the penetration.

## Introduction

**Figure 1:**
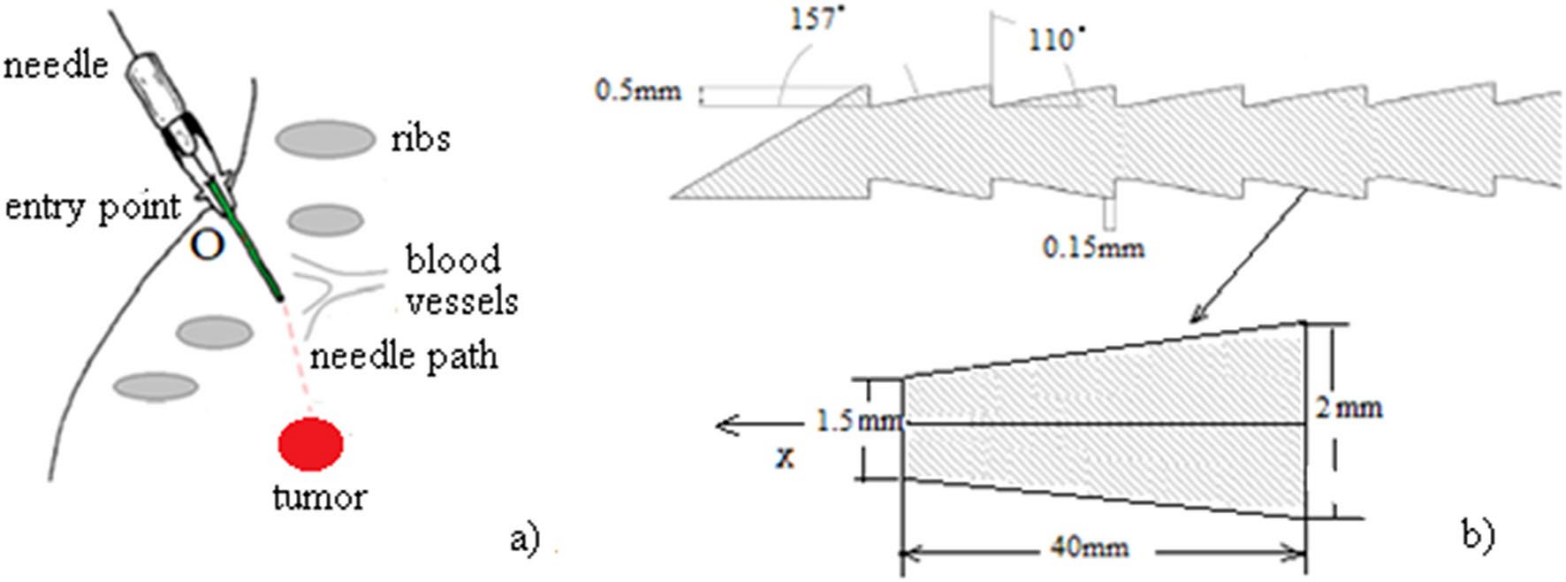
(**a**) The suggested trajectory towards the liver tumor; (**b**) Honeybee barbed needle inspired from^1,2^.

By suggesting the flexible bee needles as useful tools to transport drugs into the liver tumors, the needle navigation performance enhances^1,2^. We refer to the insertion trajectory of the needle which should avoid the ribs, blood vessels, and other organs to protect the liver^3–6^ (Fig. 1a). The bee needle has the advantage to reduce the insertion forces and to ensure small tissue deformations. The literature reports a number of interesting papers on the surgical needle navigation into the liver^7–9^. The bee needle is displayed in Fig. 1b. The front angle has 157°, the back angle, 110°, the height $h$ is 0.5 mm, and the tip thickness $b$ is 0.15 mm.

Another topic refers to the collision free trajectory of the needle to the target. This topic requires experience in imaging the tumor location, in the liver structure and in the microstructural interaction between the needle and the liver. Elastic properties of the liver, minimum execution time, minimum energy of the needle navigation, load carrying capacity are some topics of interest.

The needle flexibility is essential for a good precision in the handling. Important concentration in strain and stress and the topological changes of the liver are not to be neglected during the needle navigation towards the tumor^10–13^. Details of the forces during needle insertion into the liver are find in^14^, the real time collision detection for virtual surgery in^15^ and the minimal hierarchical collision detection in^16^. Optimization is required to modify the needle trajectory in order to protect the liver^17,18^, to manage the tumor risk^19^, and to change the robot architecture^20–22^. The inverse sonification problem for capturing hardly detectable details in a medical image is treated in^23^, and the control in^24–27^.

**Figure 2:**
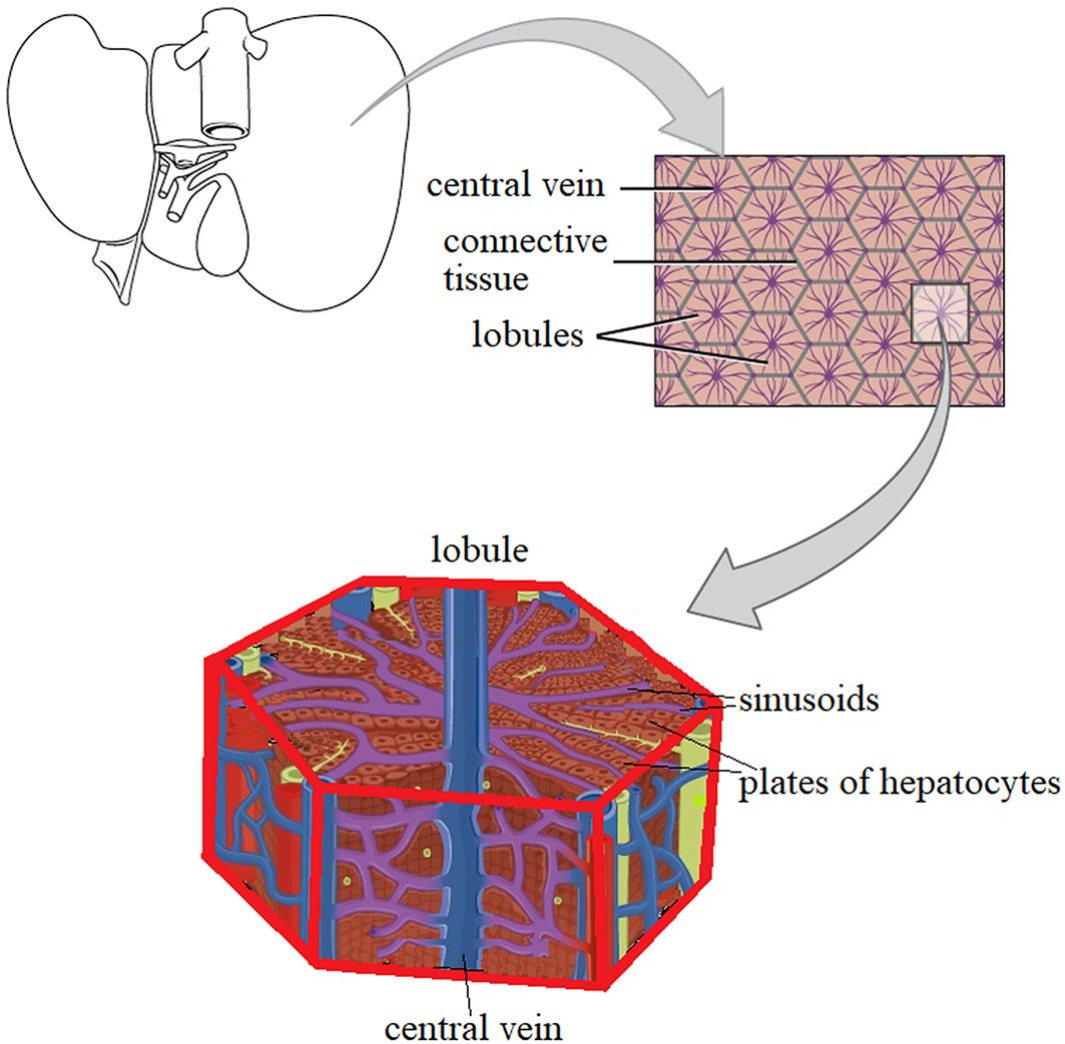
Representation of the hepatic lobule—basic functional unit of the liver.

Microscopic investigation of the human liver offers details of its microanatomy with emphases to the granular, fibrillar components and irregular solid–fluid interfaces^28–30^. The basic functional unit of the liver is the hepatic lobule which comprises a hexagonal and a portal triad-portal vein, hepatic artery, bile duct^31,32^. Lobuli form a two layers membrane with internal space of 100 A and the cellular elements with twisted, spiraling fibers braided into the helical and screw-shaped gaps (pores) of 40–100 µm in size^33–36^ (Fig. 2).

A straight line for the needle trajectory is typically used in the treatment of the liver tumors. But avoiding the obstacles and reaching the regions currently inaccessible using straight line trajectory require the motion planning algorithm for flexible needle insertion with integrative models of human liver^37^ with knowledge of the interaction forces raised during needle insertion^38^. The feedback force from the needle can be formulated as the gradient of the potential energy of the soft tissue based on particle constraint^39^. The trajectories inside the liver must be recorded by a camera to compare with the simulation trajectories in order to reduce the errors between the experimental and simulation trajectories less than 0.8 mm^40^.

Development of a robotic system and control algorithms is discussed in^41^ with different topics related to needle steering. Mechanics-based models are adapted from beam theories^42–44^. In these models, the fact that the needle deflection and tissue deformation are coupled is considered. 3D-needle shape reconstruction with an array of fiber brag grating sensors is developed in^45^ and inventions were reported for guiding devices for needle placement and performing the percutaneous computed tomography-guided surgical activity^46–48^.

Two challenges persist until now: what kind of material the liver is made of, and which is the interaction between the needle and the liver. The present paper puts together results on the elastic properties of the human liver, deformation of the needle and the liver, respectively.

Investigations on the human liver confirm a chiral (noncentrosymmetric) behavior providing evidence of isotropy with respect to the coordinate rotations but not with respect to inversions. The Cosserat (micropolar) elasticity is the appropriate theory that recognizes and describe the rotation of the cellular components as well as the translation, the couple per unit area as well as force per unit area of the hepatic membranes, the size effect in tension and bending, and the stress concentration near discontinuities^49–54^. It should be added that these features cannot be described by conventional elastic theories. The chirality leads to a vibrational amplification of the displacements and stresses which can be explained by a suitable adaptation of the liver dynamics to the attractive and repulsive forces. The chirality-triggered oscillations suggest that the linearity is preserved at the microscopic level, while becoming strongly nonlinear to the macroscopic scale.

## Deformation of the needle

**Figure 3:**
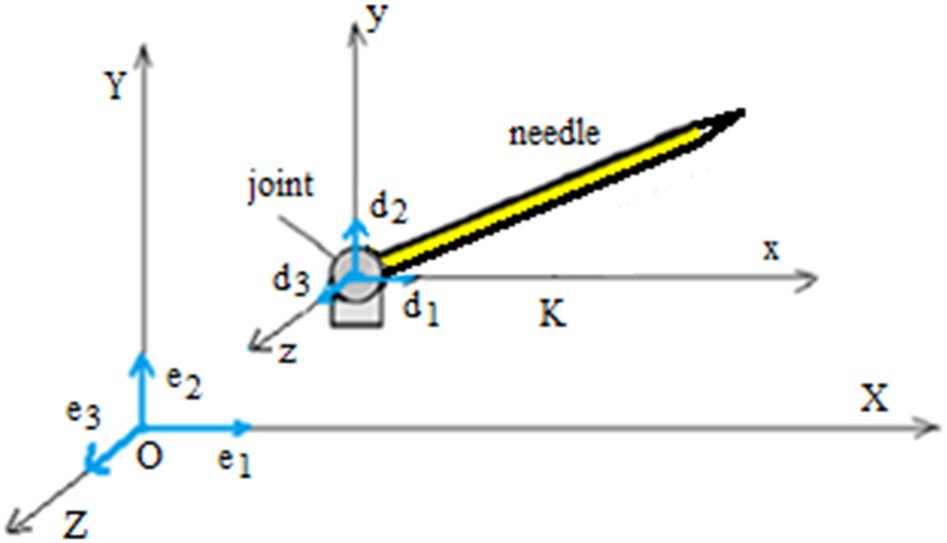
Schematic of the needle with the Lagrange coordinate system $OXYZ$ and the Euler coordinate system $oxyz$ attached.

In this research, the common focus of a serial surgical robot composed of a revolute joint and a flexible needle, was set to a reference Lagrange frame $(X,Y,Z)$ of base vectors $(e_{1} ,e_{2} ,e_{3} )$ and origin $O$ in the entry point of the skin (Fig. 3). The Euler frame $K(x,y,z)$ with origin in the joint and the base vectors $(d_{1} ,d_{2} ,d_{3} )$ is attached to the needle. The angle between the flexible arm and axis $x$ is ${\uptheta }$. Bending and torsion of the needle are described by the strain functions $(u_{1} ,u_{2} ,u_{3} )$. The robot has $f$ degrees of freedom $f = f_{r} + f_{e}$, where $f_{r} =$ 1 is the generalized coordinate of the rigid system and $f_{e}$ = 3 are the degrees of freedom of the flexible needle.

According to the previous studies of Munteanu and Donescu^55,56^, the orientation of the Euler axes relative to the Lagrange axes are expressed by Euler angles $\upsilon ,\psi$ and $\varphi$

1 $$  \begin{aligned}   d_{1}  =  &\, ( - \sin \psi \sin \varphi  + \cos \psi \cos \varphi \cos \upsilon )e_{1}  + \; + \,(\cos \psi \sin \varphi  + \sin \psi \cos \varphi \cos \upsilon )e_{2}  - \sin \upsilon \cos \varphi \, e_{3} , \\    d_{2}  =  &\, ( - \sin \psi \cos \varphi  - \cos \psi \sin \varphi \cos \upsilon )e_{1}  +\,  + (\cos \psi \cos \varphi  - \sin \psi \sin \varphi \cos \upsilon )e_{2}  + \sin \upsilon \sin \varphi e_{3} ,{\mkern 1mu}  \\    d_{3}  =  &\, \sin \upsilon \cos \psi e_{1}  +  \sin \upsilon \sin \psi e_{2}  + \cos \upsilon e_{3}. \\  \end{aligned}  $$

Strain functions $(u_{1} ,u_{2} ,u_{3} )$ measure the bending and torsion of the needle as

2 $$ \begin{aligned} u_{1} =& \, \upsilon^{\prime}\sin \varphi - \psi^{\prime}\sin \upsilon \cos \varphi , \hfill \\ u_{2} =& \, \upsilon^{\prime}\cos \varphi + \psi^{\prime}\sin \upsilon \sin \varphi , \hfill \\ u_{3} =& \, \varphi^{\prime} + \psi^{\prime}\cos \upsilon \,, \hfill \\ \end{aligned} $$

where $\left( ^{\prime} \right)$ means the partial differentiation with respect to *s*—the coordinate along the central line of the needle.

Especially to our problem, $u_{1}$ and $u_{2}$ measure the bending of the needle, and the function $u_{3}$ measures the torsion of the needle. Therefore, $u_{1}$ and $u_{2}$ are components of the curvature of the central line denoted by $\kappa$ corresponding to the planes $(yz)$ and $(xz)$

3 $$ \kappa^{2} = u_{1}^{2} + u_{2}^{2} = \upsilon^{{\prime}{2}} + \psi^{{\prime}{2}} \sin^{2} \upsilon , $$

while $u_{3}$ is the torsion of the needle denoted by $\tau$

4 $$ u_{3} = \tau = \varphi^{\prime} + \psi^{\prime}\cos \upsilon . $$

In this way, the needle is rigid along the tangential direction and the total length of the needle $l$ is invariant, the ends being fixed by external forces.

The link between the position vector $r = (x,y,z)$ and unit tangential vector $d_{3}$ is obtained as $r = \int\limits_{0}^{s} {d_{3} } {\text{d}}s$, or

5 $$ x(s) = \int\limits_{0}^{s} {\cos \psi \sin \upsilon {\text{d}}s} ,\;y(s) = \int\limits_{0}^{s} {\sin \psi \sin \upsilon {\text{d}}s} ,\;z(s) = \int\limits_{0}^{s} {\cos \upsilon {\text{d}}s} . $$

To write the equations which describe the needle deformation, we introduce the inertia of the needle characterized by the functions

6 $$ (\rho_{0} A_{0} )(s),(\rho_{0} I_{{\mathbf{1}}} )(s),(\rho_{0} I_{2} (s), $$

where $\rho_{0}$ is the mass density per unit volume, *A*_0_ the area of the cross section, $I_{1} ,I_{2}$ are geometrical moments of inertia around the axis, which is perpendicular to the central axis and respectively around the central axis.

The exact set of equations of the needle with the ends fixed by the force $F = - \lambda$ with $\lambda = (\lambda_{1} ,\lambda_{2} ,\lambda_{3} )$ becomes

7 $$ - \rho \ddot{r} - \lambda^{\prime} = 0, $$

8 $$ k_{1} (\dot{\psi }^{2} \sin \upsilon \cos \upsilon - \ddot{\upsilon }) - k_{2} (\dot{\varphi } + \dot{\psi }\cos \upsilon )\,\dot{\psi }\sin \upsilon - A(\psi^{^{\prime}2} \sin \upsilon \cos \upsilon - \upsilon^{\prime\prime}) + C(\varphi^{\prime} + \psi^{\prime}\cos \upsilon )\psi^{\prime}\sin \upsilon - \lambda_{1} \cos \upsilon \cos \psi - \lambda_{2} \cos \upsilon \sin \psi + \lambda_{3} \sin \upsilon = 0, $$

9 $$ - \frac{\partial }{\partial t}\{ k_{1} \dot{\psi }\sin^{2} \upsilon + k_{2} (\dot{\varphi } + \dot{\psi }\cos \upsilon )\,\cos \upsilon \} + \frac{\partial }{\partial s}\{ A\psi^{^{\prime}2} \sin^{2} \upsilon + C(\varphi^{\prime} + \psi^{\prime}\cos \upsilon )\cos \upsilon \} + \lambda_{1} \sin \upsilon \sin \psi - \lambda_{2} \sin \upsilon \cos \psi = 0, $$

10 $$ - k_{2} \frac{\partial }{\partial t}(\dot{\varphi } + \dot{\psi }\cos \upsilon ) + C\frac{\partial }{\partial s}(\varphi^{\prime} + \psi^{\prime}\cos \upsilon ) = 0, $$

where *A* and *C* are the bending stiffness and respectively the torsional stiffness of the needle, related to the Lame constants $\overline{\lambda }$, $\mu$ by $A = \frac{1}{4}\pi a^{4} E,\,\,\,\,\,\,C = \frac{1}{2}\pi a^{4} \mu ,\,$ where $E = \frac{{\mu (3\overline{\lambda } + 2\mu )}}{{\overline{\lambda } + \mu }}$ is the Young's elastic modulus, and *a* is the radius of the cross section of the needle, and

11 $$ \rho = A_{0} \rho_{0} = \pi a^{2} \rho_{0} \,,\,\,\,\,k_{1} = I_{1} \rho_{0} = \frac{{\pi a^{4} }}{4}\rho_{0} ,\,\,\,\,k_{2} = I_{2} \rho_{0} = \frac{{\pi a^{4} }}{2}\rho_{0} . $$

The system of Eqs. (7)–(11) is exactly solved by the cnoidal method. As a result, the closed form solutions of the Euler angles $\theta ,\psi$ and $\varphi$ are derived^55^

12 $$ \cos \upsilon = \zeta = \zeta_{2} - (\zeta_{2} - \zeta_{3} ){\text{cn}}^{{2}} (\sqrt {\frac{{|\lambda_{3} |}}{2A}(\zeta_{1} - \zeta_{3} )} (\xi - \xi_{3} ),m) = \zeta_{2} - (\zeta_{2} - \zeta_{3} ){\text{cn}}^{{2}} [w(\xi - \xi_{3} ),m], $$

with $$m = \frac{{\zeta_{2} - \zeta_{3} }}{{\zeta_{1} - \zeta_{3} }}$$ and $$w = \sqrt {\frac{{|\lambda_{3} |}}{2A}(\zeta_{1} - \zeta_{3} )} ,$$

13 $$ \psi = \frac{1}{{4(A - k_{1} v^{2} )^{2} w^{2} }}\{ - \frac{{\beta + (C - k_{2} v^{2} )\tau }}{{1 - \zeta_{3} }}\Pi [w(\xi - \xi_{3} ),\frac{{\zeta_{2} - \zeta_{3} }}{{1 - u_{3} }},m] - \frac{{\beta - (C - k_{2} v^{2} )\tau }}{{1 + \zeta u_{3} }}\Pi [w(\xi - \xi_{3} ),\frac{{\zeta_{2} - \zeta_{3} }}{{1 + u_{3} }},m]\} , $$

14 $$ \begin{aligned} \,\varphi = & - \frac{{\tau [C - A - (k_{2} + k_{1} )v^{2} ]}}{{A - k_{1} v^{2} }}\xi + \frac{1}{{4(A - k_{1} v^{2} )^{2} w^{2} }}\{ \frac{{\beta + (C - k_{2} v^{2} )\tau }}{{1 - \zeta_{3} }} \\ \, \times & \Pi [w(\xi - \xi_{3} ),\frac{{\zeta_{2} - \zeta_{3} }}{{1 - \zeta_{3} }},m] - \frac{{\beta - (C - k_{2} v^{2} )\tau }}{{1 + \zeta_{3} }}\Pi (w(\xi - \xi_{3} ),\frac{{\zeta_{2} - \zeta_{3} }}{{1 + \zeta_{3} }},m)\} , \\ \end{aligned} $$

where $\Pi (x,z,m) = \int\limits_{0}^{x} {\frac{{{\text{d}}y}}{{1 - z\,{\text{sn}}^{2} (y,m)}}}$ is the normal elliptic integral of the third kind. Functions $\zeta_{1} ,\zeta_{2} ,\zeta_{3}$ are solutions of the equation

15 $$ \frac{1}{2}\zeta^{{\prime}{2}} = a\zeta^{3} + b\zeta^{2} - a\zeta + c, $$

with

16 $$ a = - \frac{{\lambda_{3} }}{A} \ne 0,\;b = \frac{1}{2A}\left( {\gamma - \frac{{C^{2} \tau^{2} }}{A}} \right),\;c = - \frac{1}{2A}\left( {\gamma - \frac{{\beta^{2} }}{A}} \right). $$

**Figure 4:**
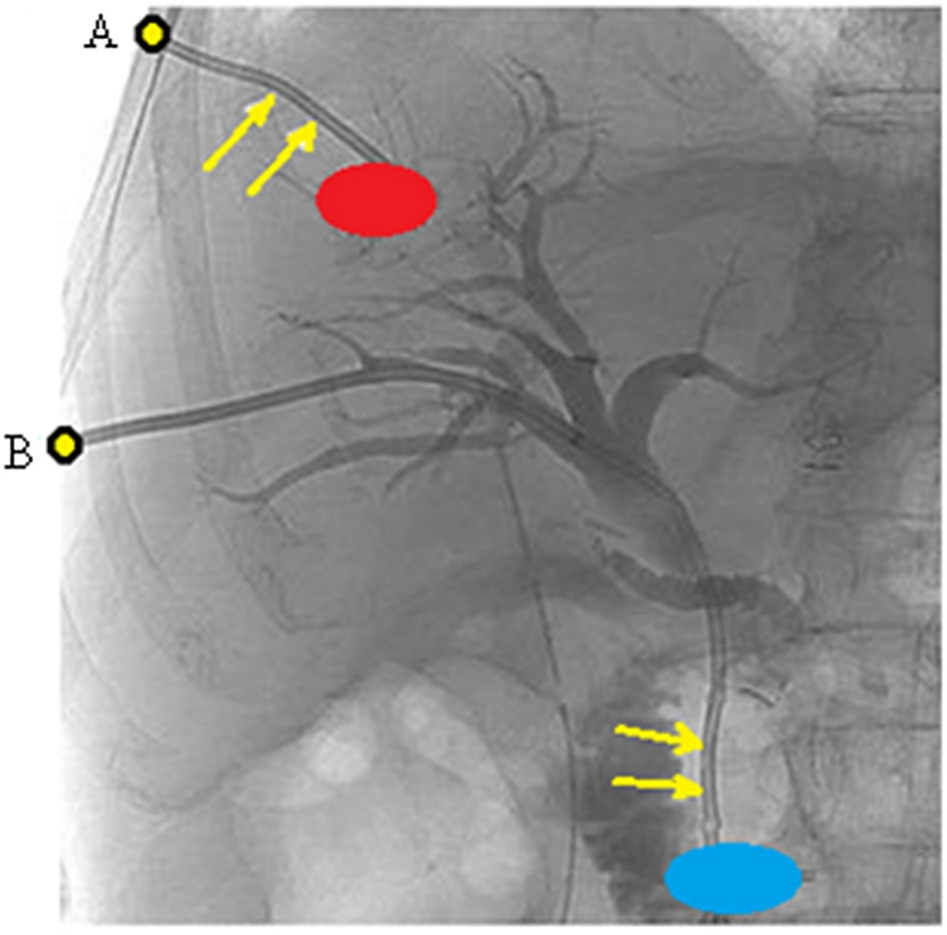
Two needle routes: the first route with a red tumor and the entry point A, and the second route longer with a blue tumor and the entry point B.

For the present study, our objective is to determine the functions which measure the bending of the needle ($u_{1}$ and $u_{2}$), and the function which measures the torsion of the needle ($u_{3}$). This can be done by (2). The strain profile of the needle is computed for two needle routes as considered in Fig. 4. For the first route, the tumor is red and the entry point is A and for the second route the tumor is blue with entry at point B.

**Figure 5:**
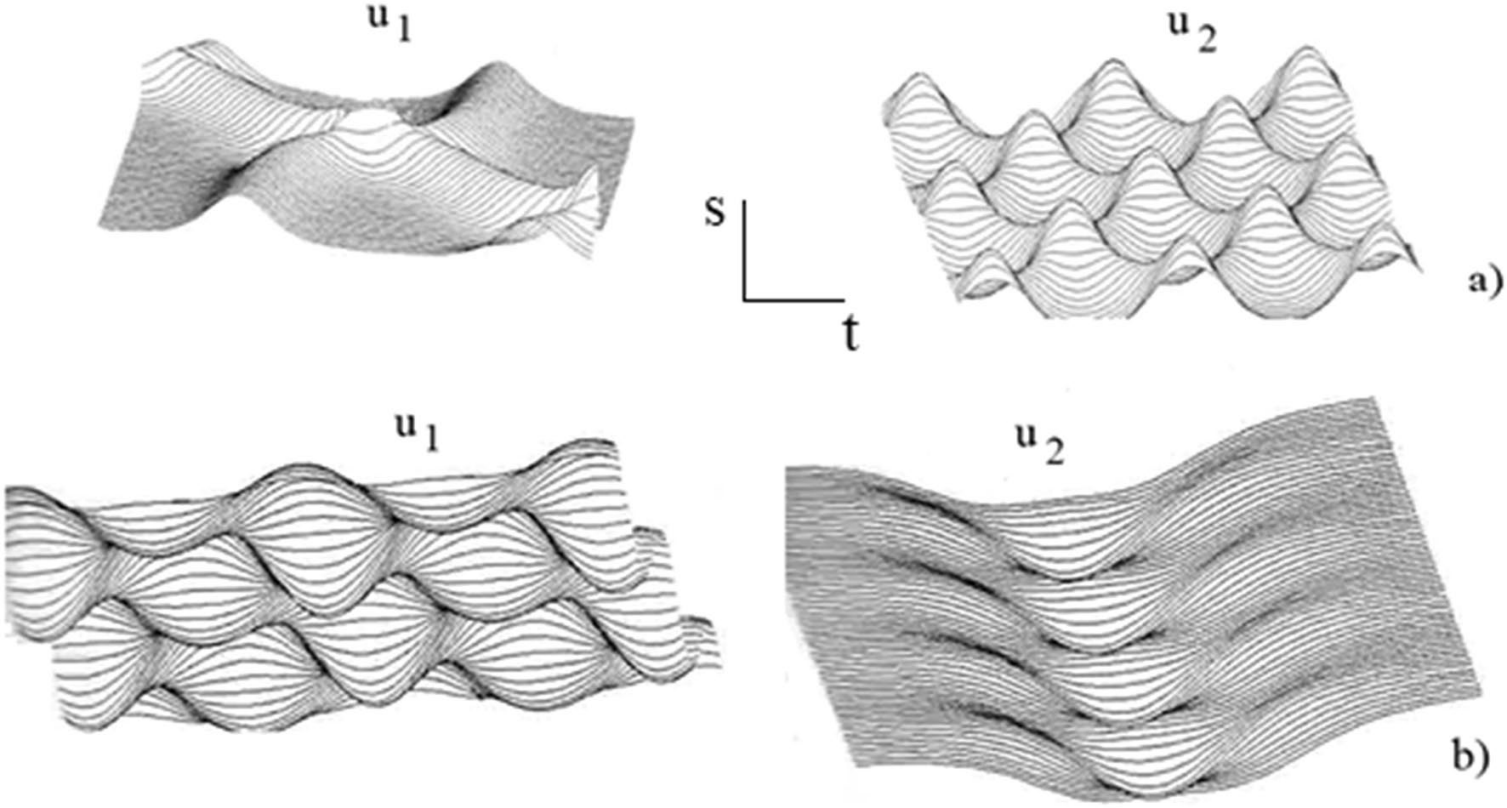
Functions $u_{1}$ and $u_{2}$ for (**a**) the first route and (**b**) the second route.

**Figure 6:**
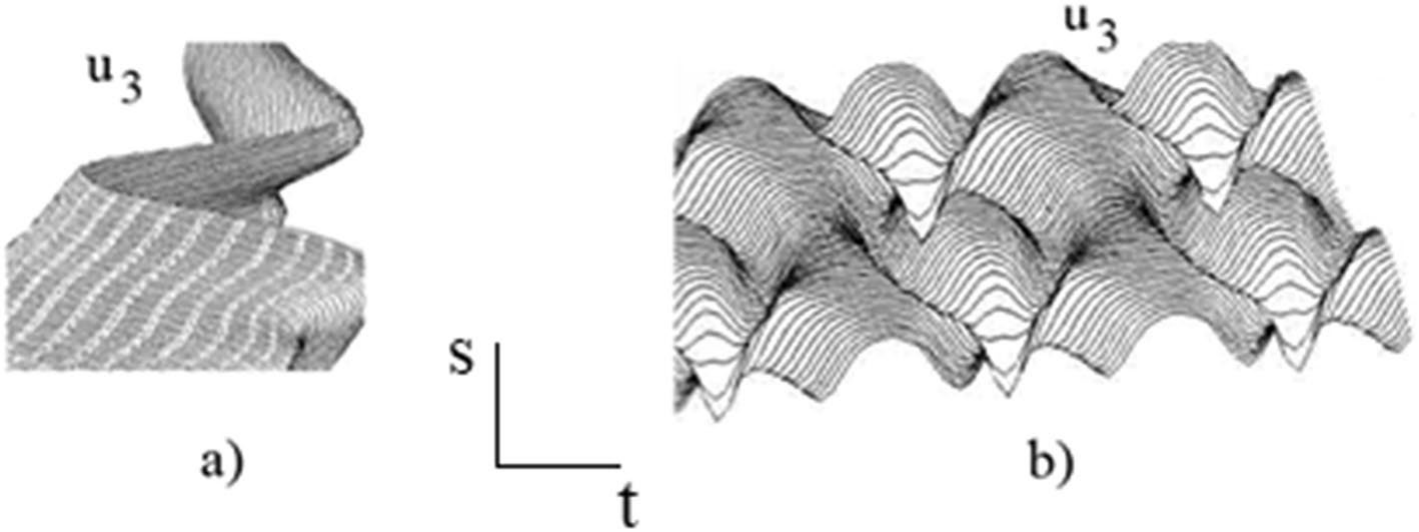
Function $u_{3}$ for (**a**) the first route and (**b**) the second route.

Figure 5 represents the bending functions $u_{1}$ and $u_{2}$ of the needle which depend on the coordinate along the central line of the needle $s$, and the time $t$ , for the route (a) and for the route (b), respectively. In a similar matter, Fig. 6 represents the torsion functions $u_{3}$ of the needle.

It is noteworthy that Figs. 5 and 6 show that the deformation of the needle depends on the needle trajectory. For both cases, the deformation of the needle is small and finite without any tendency to grow towards chaos. The strains operate in a solitonic regime in which the localized waves propagate for a long time without changes. The soliton is a localized wave with an infinite number of degrees of freedom that conserve their properties even after interaction among them, and then act somewhat like particles^55^. The basic idea is that the Eqs. (7)–(11) have unique properties that are locally preserved such as an infinite number of exact solutions expressed in terms of the Jacobi elliptic functions or the hyperbolic functions, and the simple formulae for nonlinear superposition of explicit solutions.

Degree of deformation of the honeybee barbed needle during insertion into the liver is caused by the flexibility of the needle. The main parameters that determine the flexibility of the needle are the height $h$ and the tip thickness of the needle $b$ (see Fig. 1b).

**Figure 7:**
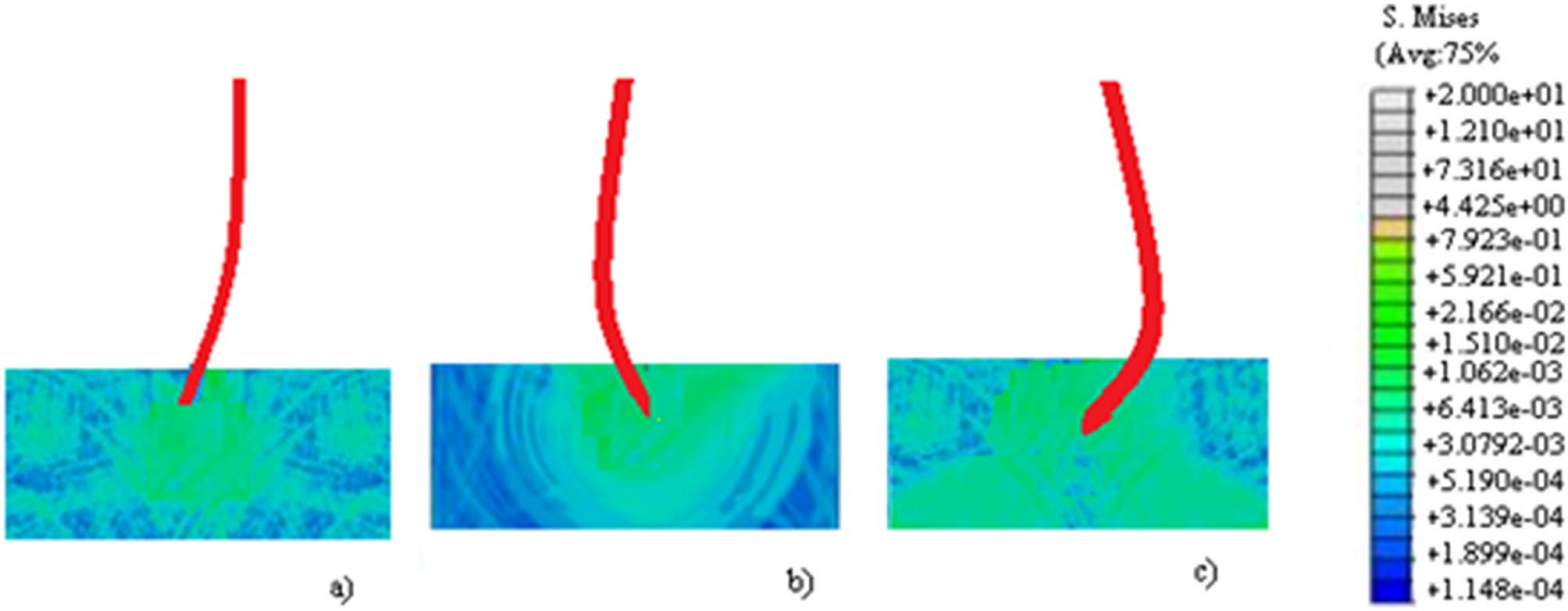
Insertion simulation results for different values of $h$ and $b$; (**a**) $h =$ 0.5 and $b =$ 0.15; (**b**) $h =$ 0.55 and $b =$ 0.2; (**c**) $h =$ 0.6 and $b =$ 0.25.

For study the effect of the needle parameters $h$ and $b$ on the stress change during the insertion, simulations are carried out under different values for these parameters, as $h =$ 0.5, 0.55, 0.6 and $b =$ 0.15, 0.2, 0.25. The simulation results are shown in Fig. 7 for $b$; a) $h =$ 0.5 and $b =$ 0.15; b) $h =$ 0.55 and $b =$ 0.2; c) $h =$ 0.6 and $b =$ 0.25.

We can see from the Fig. 7 that if the parameters $h$ and $b$ of the needle increase, the deformation of the needle in the tissue also increases. The stress order of magnitude for $h =$ 0.5 and $b =$ 0.15 is 0.01, for $h =$ 0.55 and $b =$ 0.2 is about 0.1, while for $h =$ 0.6 and $b =$ 0.25 is about 0.17. We conclude that the larger parameters $h$ and $b$ of the needle, the greater is the stress distribution.

## Elastic constants of the liver

Cosserat theory makes our work distinct from previous studies describing the elasticity of the human liver. In spite of the fact that the liver was usually modeled as an elastic medium, we noted a rich dynamic behavior of the human liver involving nonlinearity and chirality. Our findings show that the effect of the chirality leads to a vibrational amplification of the elastic response of the liver due to the needle penetration, explained by the adaptation of the liver dynamics to the attractive and repulsive forces (inter-atomic bonds). The human liver is viewed as a spatial overlap of mesoscopic subsystems in which interferences overlap or are lost, as such it naturally vibrates through overlapping the oscillations—and this result is a natural effect of chirality. Therefore, the human liver can be regarded as an elastic chiral Cosserat material (noncentrosymmetric) characterized by the fourth rank elastic constant^49–56^

17 $$ C_{ijkl} = \frac{{{\text{d}}x_{m} }}{{{\text{d}}x_{i} }}\frac{{{\text{d}}x_{n} }}{{{\text{d}}x_{j} }}\frac{{{\text{d}}x_{{\text{o}}} }}{{{\text{d}}x_{k} }}\frac{{{\text{d}}x_{p} }}{{{\text{d}}x_{l} }}C_{mnop} = ( - 1)^{4} C_{ijkl} = C_{ijkl} = \frac{{\partial^{2} V}}{{\partial \varepsilon_{ij} \partial \varepsilon_{kl} }},\quad i,j,k,l = 1,2,3, $$

where $V = \frac{U}{\Omega }$ is the potential energy of deformation per unit volume (elastic potential) of the hepatic lobule, $U$ is the total energy of the liver, $\Omega$ is the volume of the hepatic lobule, and $\varepsilon_{ij}$ is the Lagrangian strain tensor, and

18 $$ \frac{\partial }{{\partial \varepsilon_{ij} }} = \frac{1}{2}\left( {X_{i} \frac{\partial }{{\partial x_{j} }} + X_{j} \frac{\partial }{{\partial x_{i} }}} \right), $$

with $X_{i}$ the material or Lagrangian coordinates and $x_{i}$ the Eulerian coordinates.

After that, the potential theory^45^ is defined by four terms: the free-electron energy $U_{fe}$, then electrostatic Coulomb energy $U_{es}$ which is often called the Mandelung energy, the band-structure energy $U_{bs}$ and the Born–Mayer ion-core repulsive energy $U_{r}$^57–62^

19 $$ U = U_{fe} + U_{es} + U_{bs} + U_{r} . $$

Jankowski and Tsakalakos^59^ advanced that $U_{r}$

20 $$ U_{r} = \frac{1}{2}\tilde{\alpha }\sum\limits_{n} {\exp ( - \tilde{\beta }R^{(n)} )} , $$

is the predominant term for the evaluation of the elastic constants. The sum is extended to all nearest neighbors points of coordinates $(X,Y,Z)$ located at distances $R^{(n)}$, $R = \sqrt {X_{{}}^{2} + Y_{{}}^{2} + Z_{{}}^{2} }$ with respect to the green atom from Fig. 7, $R^{(n)}$, $\tilde{\alpha }$ is the parameter of the repulsive energy and $\tilde{\beta }$ is the repulsive range parameter^57^. The $\tilde{\alpha }$ is measured in Ryd (Rydberg) 1 Ryd = 13.6 eV = 2.092 $\times$ 10 $^{ - 21}$ J, and $\tilde{\beta }$ in atomic units [ua]. These parameters are evaluated from a genetic algorithm that use the experimental value of the elastic modulus of the liver $E =$ 5.9359 Pa and Poisson’s ratio $\nu =$ 0.49 available in^58^.

The aim of the inverse problem is to use the difference between the experimental and theoretical values of the Young’s modulus and the Poisson’ratio to provide a procedure that leads to the least disaccord between predictions and experimental observations. We consider that $\tilde{\alpha }$ and $\tilde{\beta }$ are approximated by polynomials of five degree $P_{i} (b_{6i - 5} , \ldots, b_{6i} )$, $i = 1,2, \ldots, 7$, characterized by coefficients $b_{j}$, $j = 1,2, \ldots, 42$. To calculate $P_{i}$, $i = 1,2,...7$ from experimental data, an objective function $\Im$ is chosen to measure the agreement between theoretical and experimental data

$$ \Im {(}P{)} = { 42}^{{ - {1}}} \sum\limits_{j = 1}^{42} {4^{ - 1} \sum\limits_{{{\text{i}} = {1}}}^{{2}} {{\text{[z}}_{i} {(}b_{j} {)} - z_{i}^{\exp } {(}b_{j} {)]}^{2} } } , $$

where $$z_{i} {(}b_{j} {)}$$, $$i = 1,2$$ are the predicted values of $$\tilde{\alpha }$$ and $$\tilde{\beta }$$, and $$z_{i}^{\exp }$$, $$i = 1,2$$, are the experimental values of $$\tilde{\alpha }$$ and $$\tilde{\beta }$$. The functions $$P_{i}$$, $$i = 1,2, \ldots ,7$$, are estimated by a genetic algorithm. This algorithm assures an iteration scheme that guarantees a closer correspondence of predicted and experimental values of $$\tilde{\alpha }$$ and $$\tilde{\beta }$$ at each iteration. A binary vector with 42 genes representing the real values of the parameters $$b_{j}$$, $$j = 1,2 \ldots 42$$, is used. The length of the vector is six places after the decimal point. The domain of parameters $$b_{j} \in$$$$[ - a_{j} ,a_{j} ]$$ with length $$2a_{j}$$ is divided into a least 15,000 equal size ranges. That means that each parameter $$b_{j}$$, $$j = 1,2 \ldots 42$$, is represented by a gene (string) of 22 bits $$2^{21} < 3000000 \le 2^{22}$$. One individual consists of the row of 42 genes, that is, a binary vector with 22 $$\times$$ 42 components given by $$b_{21}^{(1)} b_{20}^{(1)} \ldots b_{0}^{(1)} b_{21}^{(2)} b_{20}^{(2)} \ldots b_{0}^{(2)} \ldots b_{21}^{(42)} b_{20}^{(42)} \ldots b_{0}^{(42)}$$. From one generation to the next the genetic algorithm usually decreases the objective function of the best model. The starting population is usually randomly generated. Then, new descendant populations are iteratively created with the goal of an overall objective function decrease from generation to generation. Each new generation is created from the current one by the main operations: selection, crossover and reproduction, mutation and fluctuation. The alternation of generations is stopped when convergence is detected. If no convergence the iteration process continues until the specified maximum number of generations is reached.

We report here the results of the genetic algorithm. The unknown $\tilde{\alpha }$ and $\tilde{\beta }$ are obtained after 31 iterations. The results of the genetic algorithm are $\tilde{\alpha } \times 10^{6} = 0.19$ Ryd and $\tilde{\beta } =$ 10.22 ua.

Describe the constitutive equations for the isotropic centrosymmetric Cosserat solids as follows^49–53^

21 $$ \sigma_{kl} = \lambda e_{rr} \delta_{kl} + (2\mu + \kappa )e_{kl} + \kappa \varepsilon_{klm} (r_{m} - \varphi_{m} ), $$

22 $$ m_{kl} = \alpha \varphi_{r,r} \delta_{kl} + \beta \varphi_{k,l} + \gamma \varphi_{l,k} , $$

where $\sigma_{kl}$ is the Cauchy stress tensor, $m_{kl}$ is the couple stress tensor, $e_{kl} = \frac{1}{2}(u_{k.l} + u_{l,k} )$ is the macrostrain strain tensor, $u$ is the displacement vector and $\varepsilon_{klm}$ is the Levi–Civita permutation tensor. Further, the microrotation vector $\varphi_{k}$ is distinct from the macrorotation vector $r_{k} = \frac{1}{2}\varepsilon_{klm} u_{m,l}$, i.e. $\varphi_{k}$ refers to the rotation of points themselves, while $r_{k}$ refers to the rotation associated with movement of nearby points. The comma denotes differentiation with respect to spatial coordinates and a superposed dot indicates the time rate.

Involved elastic constants are the Lame elastic constants $\lambda$, and $\mu$, the Cosserat rotation modulus $\kappa$, and the first, second and third microrotation constants $\alpha ,\beta ,\gamma$.

For a healthy liver without tumors, the estimates of the elastic constants $\lambda ,\mu ,\kappa ,\alpha ,\beta$ and $\gamma$ are shown in Table 1.

**Table 1:** Estimates for elastic constants in the ($X,Y,Z$) coordinates as a function of strain.

Strain	$\lambda$[Pa]	$\mu$[Pa]	$\kappa$[Pa] $\times 10^{2}$	$\alpha$ [N] $\times 10^{4}$	$\beta$[N] $\times 10^{4}$	$\gamma$[N] $\times 10^{4}$
− 0.040	97.7806	2.1033	459	2.0976	12.0833	2.0865
− 0.036	97.7703	2.0876	432	2.0878	12.0775	2.0814
− 0.032	97.7657	2.0456	401	2.0502	12.0656	2.0800
− 0.028	97.7587	1.9981	396	2.0481	11.9961	1.9976
− 0.024	97.7498	1.9978	388	1.9934	11.9908	1.9974
− 0.020	97.7480	1.9964	387	1.9914	11.9904	1.9969
− 0.016	97.7398	1.9956	381	1.9897	11.9895	1.9958
− 0.012	97,7341	1.9933	374	1.9883	11.9891	1.9913
− 0.008	97.7034	1.9928	371	1.9878	11.9792	1.9900
− 0.004	97.6351	1.9925	366	1.9865	11.9725	1.9895
0.000	97.6037	1.9919	354	1.9849	11.9719	1.9819
0.004	97.5939	1.9909	351	1.9839	11.9709	1.9809
0.008	97.5744	1.9874	342	1.9814	11.9704	1.9776
0.012	97.3901	1.9832	340	1.9802	11.9695	1.9772
0.016	96.8372	1.9802	339	1.9782	11.9692	1.9707
0.020	96.6333	1.9745	336	1.9735	11.9655	1.9705
0.024	96.4403	1.9698	332	1.9718	11.9598	1.9688
0.028	96.3012	1.9453	329	1.9653	11.9553	1.9552
0.032	95.7854	1.9245	324	1.9625	11.9545	1.9265
0.036	95.7840	1.9203	322	1.9603	11.9409	1.9213
0.040	97.7737	1.9189	321	1.9518	1.9408	1.9177

It is interesting to see from the Table 1 that the elastic constants of liver reduces all the way when the strain increases from negative (compression) to zero and then to positive(extension) values. This is explained by the dependency of the liver elasticity on the size of the inhomogeneities and the surface/interface stress at the micro-scale.

Finally, the results show that even small strains can affect the values of the elastic constants. The increase in the strain reduces the value of the elastic constants.

## Deformation of the liver during the needle insertion

The deformation of the needle is coupled with the deformation of the liver. To evaluate the liver deformation, the connection between the needle and the liver is modeled as a spring layer with a very small thickness^63–65^. The tractions are continuous but displacements can be discontinuous across the layer.

The evaluation of the liver deformation is simplified by discretization of the interface between the needle and the liver, into tiny homogeneous cells^65^. Across this grid of points, a network of springs is introduced to ensure that the behaviour inside each component is elastic and, in the case of perfect contact interfaces, a perfect contact among different components.

Figure 9 represents a 2D spring model with a generic point $O$ the tip of the needle. The nearest neighbours in the liver are labelled from 1 to 8. It is thus possible to obtain the iteration equations for the deformation of the liver starting from the deformation of the needle.

A Cartesian coordinates system $(x,y,z)$ is attached to the human liver. The needle moves in the direction of the $z$-axis, in the origin of the cartesian coordinate. The concentrated needle force has a magnitude $F = F_{0} \delta (x)\delta (y)\delta (t)$. For simplicity without loss of generality we consider that $\tau_{1} = \tau_{2} = \cdots = \tau_{8} = F_{0}$. Also, we assume the particular case in which all quantities depend only on $x$ and $z$.

To this end, the constitutive relations (21) and (22) are completed with the kinematic description which includes the microrotations $\varphi_{k}$, $k = 1,2,3$, as independent degrees of freedom in addition to the usual displacements $u_{i}$, $i = 1,2,3$. The transfer of loading between points in the liver is achieved through the couple stresses $m_{ij}$, $i,j = 1,2,3$ and the classical Cauchy stresses $\sigma_{ij}$, $i,j = 1,2,3$.

The equilibrium equations are in the absence of body forces and body couples

23 $$ \sigma_{ji,j} = 0, $$

24 $$ m_{ji,j} + \varepsilon_{jk} \sigma_{jk} = 0,\quad i,j,k = 1,2,3. $$

Define the kinematic variables as the strain tensor

25 $$ \varepsilon_{ij} = \frac{1}{2}(u_{j,i} + u_{i,j} ),\quad i,j = 1,2,3, $$

and the strain gradient tensor

26 $$ \eta_{ijk} = \varepsilon_{jk,i} = \eta_{ikj} ,\quad i,j,k = 1,2,3. $$

As we said before, the basic functional unit of the liver is the hexagonal hepatic lobule which comprises the portal triad-portal vein, hepatic artery, bile duct.

The solutions in displacements $u_{1} ,u_{2}$ and $u_{3}$ of the Eqs. (23)–(26) are taken under the form

27 $$ u(x,t) = z_{lin} (x,t) + z_{nonlin} (x,t), $$

where the first term $z_{lin}$ represents a linear superposition of cnoidal functions and the second term $z_{nonlin}$, a nonlinear superposition of the cnoidal functions^55^

28 $$ z_{cn} {(}x,t{)} = 2\sum\limits_{m = 1}^{n} {\alpha_{m} {\text{cn}}^{2} {(}k_{mj} x_{j} - C_{m} t{)}} ,\quad j = 1,2,3, $$

29 $$ z_{{\text{int}}} (x,t) = \frac{{\sum\limits_{m = 1}^{n} {\gamma_{m} {\text{cn}}^{2} {(}k_{mj} x_{j} - C_{m} t{)}} }}{{1 + \sum\limits_{m = 1}^{n} {\lambda_{m} {\text{cn}}^{2} {(}k_{mj} x_{j} - C_{m} t{)}} }},\quad j = 1,2,3. $$

**Figure 8:**
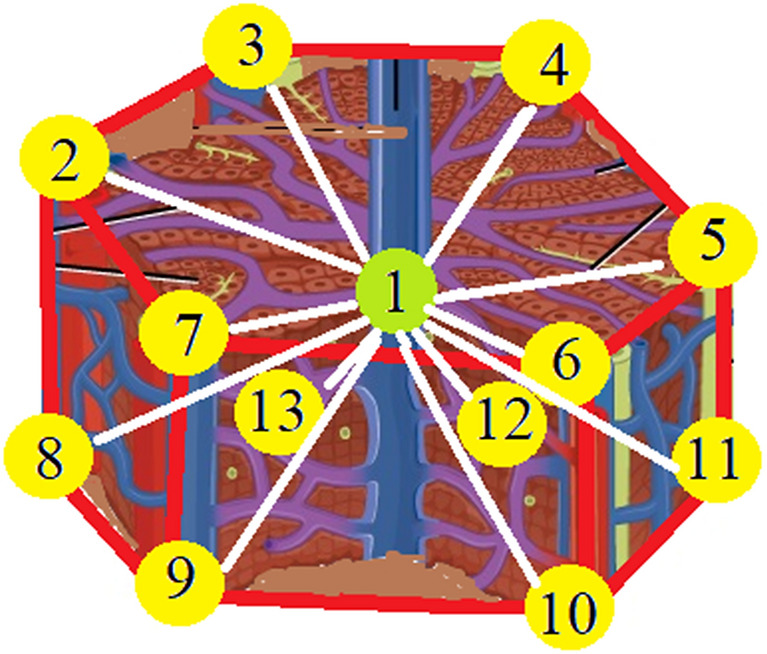
Representation of the twelve nearest neighbors of the green atom.

**Figure 9:**
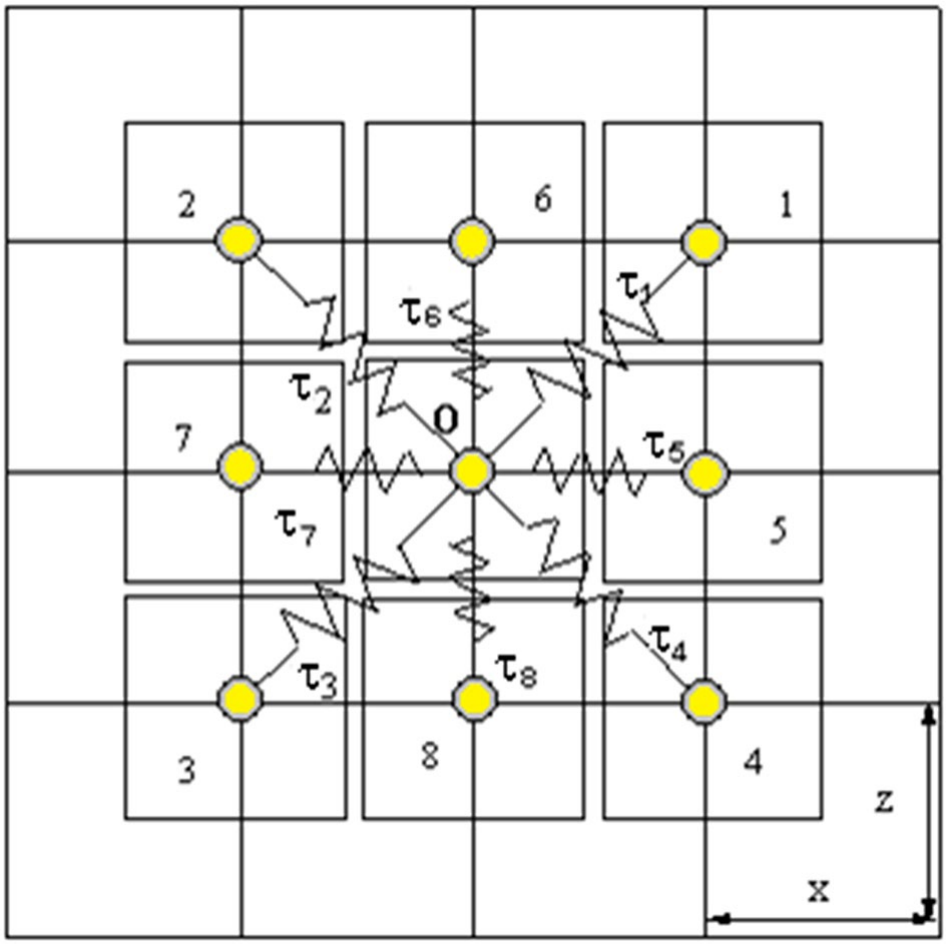
2D spring model for the interface between the tip of the needle and the liver^65^.

The analytic expressions of $U_{1} = \frac{{u_{1} }}{{F_{0} }}$, $U_{2} = \frac{{u_{2} }}{{F_{0} }}$ and $U_{3} = \frac{{u_{3} }}{{F_{0} }}$ are available once the displacements $u_{1} ,u_{2}$ and $u_{3}$ are specified. Variation of displacements $U_{1} ,U_{2}$ and $U_{3}$ with respect to $x$ for the human liver is presented in Fig. 8. Variations of the normal stresses $T_{13} = \frac{{\sigma_{13} }}{{F_{0} }}$,$T_{31} = \frac{{\sigma_{31} }}{{F_{0} }}$,$T_{32} = \frac{{\sigma_{32} }}{{F_{0} }}$ and $T_{33} = \frac{{\sigma_{33} }}{{F_{0} }}$ with respect to $x$ are presented in Fig. 9

**Figure 10:**
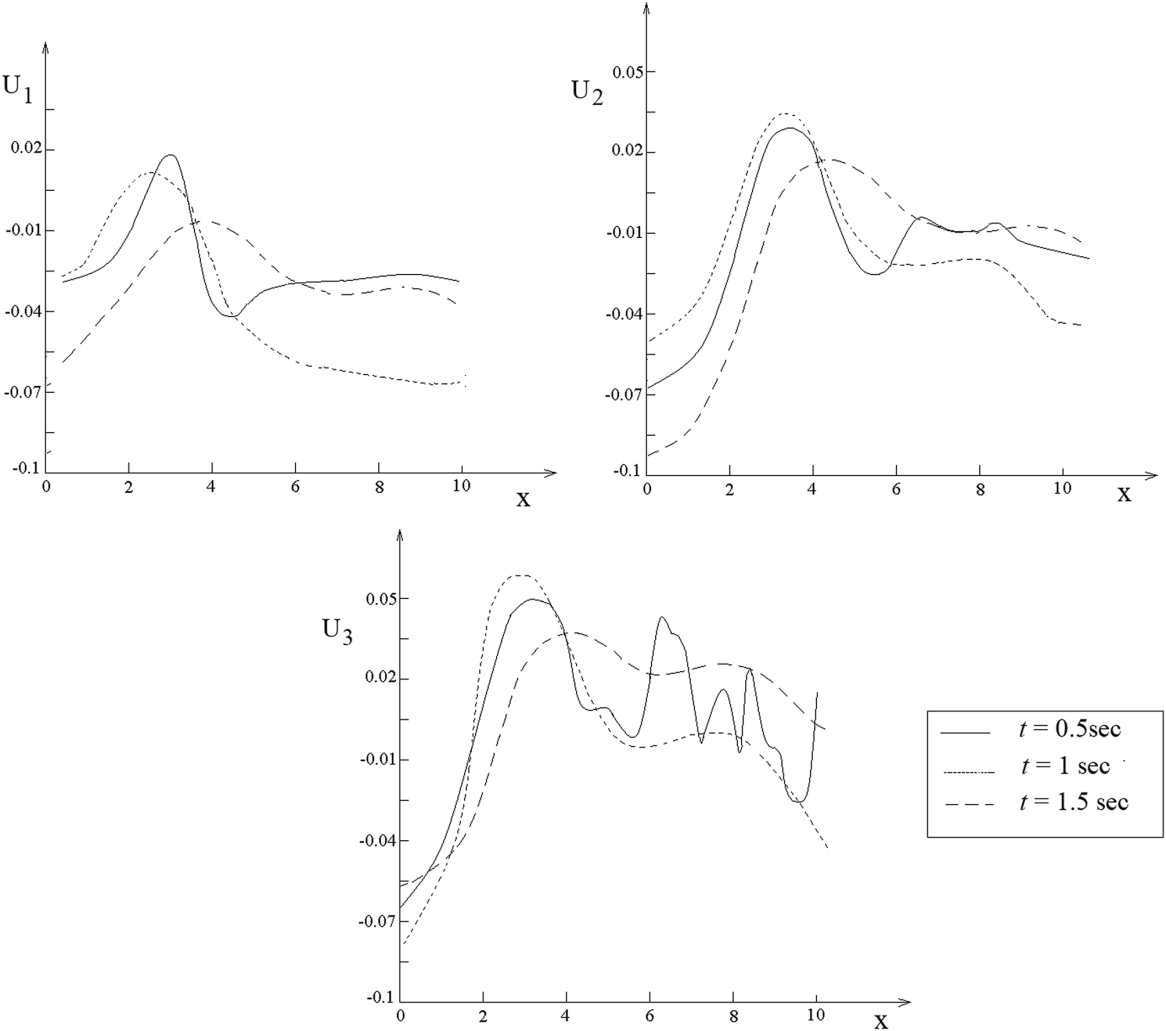
Plots of $U_{1} ,U_{2}$ and $U_{3}$ with respect to $x$.

**Figure 11:**
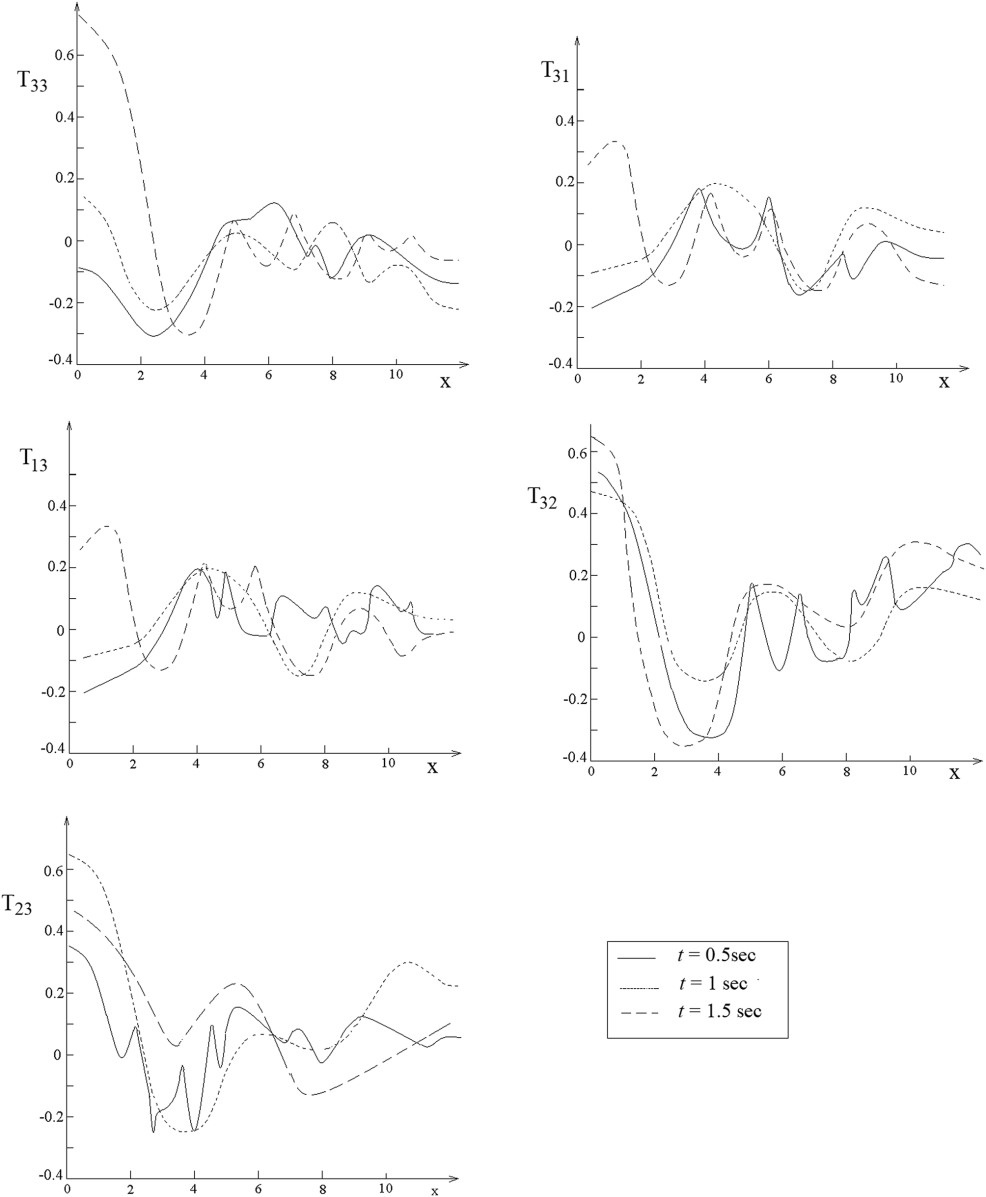
Plots of $T_{13} ,T_{23}$ and $T_{33}$ with respect to $x$.

Graphs from Figs. 10 and 11 illustrate an oscillatory behavior. After 1.5 s the graphs do not change significantly, this means that the soliton functions with which these solutions are expressed are stabilized as form and identity. The oscillatory phenomenon is a result of the emission of acoustic signals rich in ultrasound components through which the liver atoms communicate between them to balance the liver state. The role of vibrations in the strain and the stress fields depends on the ability of the liver to respond to loading conditions by making use of the vibratory communication between tissue and the needle. This communication is made by means of excitation signals rather that the response signals alone. To our knowledge, this vibratory communication of the human liver with the needle has not previously been recorded.

**Figure 12:**
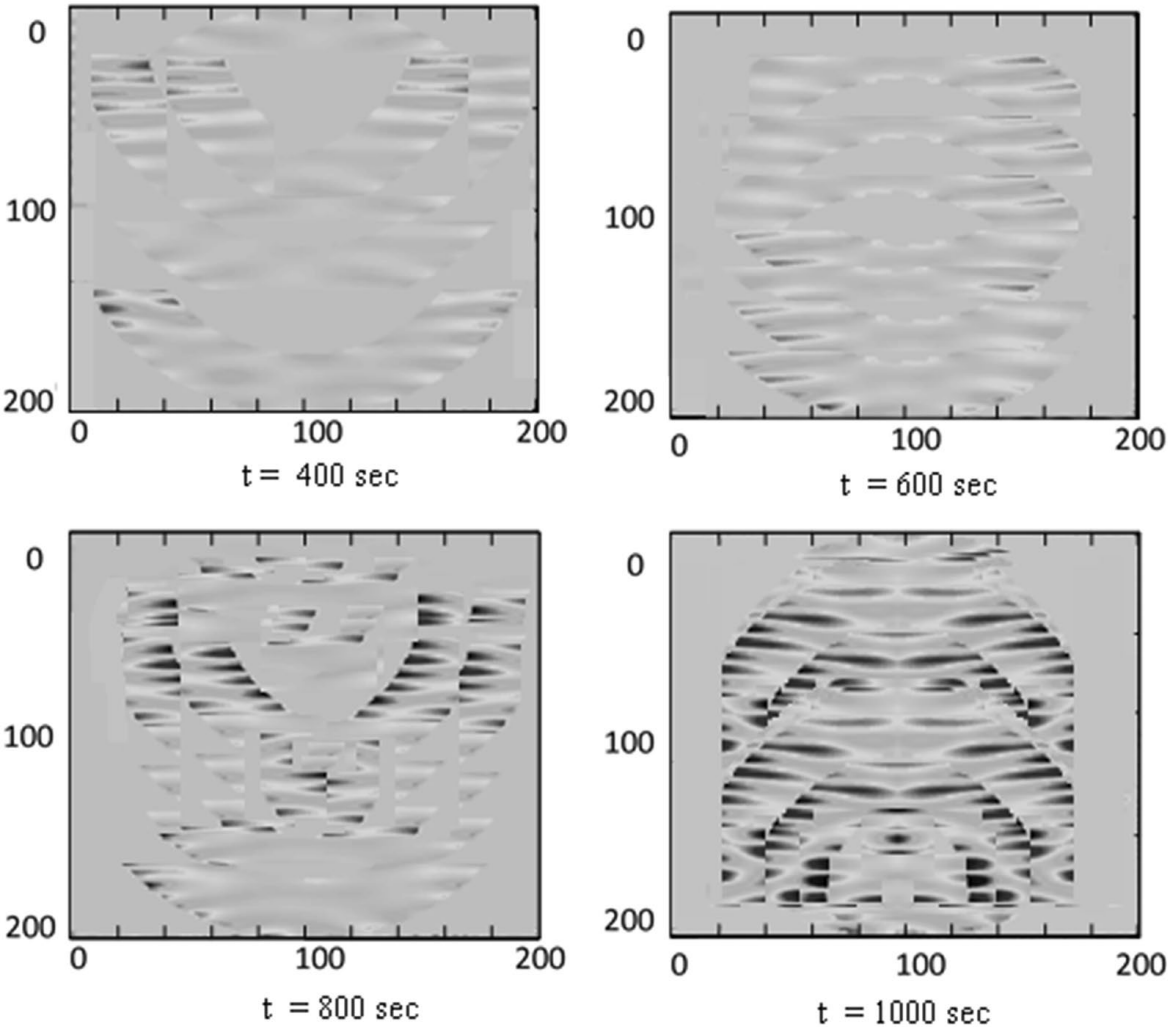
Snapshots at various time of the amplitude of the stress in the tip of the needle.

Snapshots at various time of the amplitude of the stress in the tip of the needle are shown in Fig. 12.

**Figure 13:**
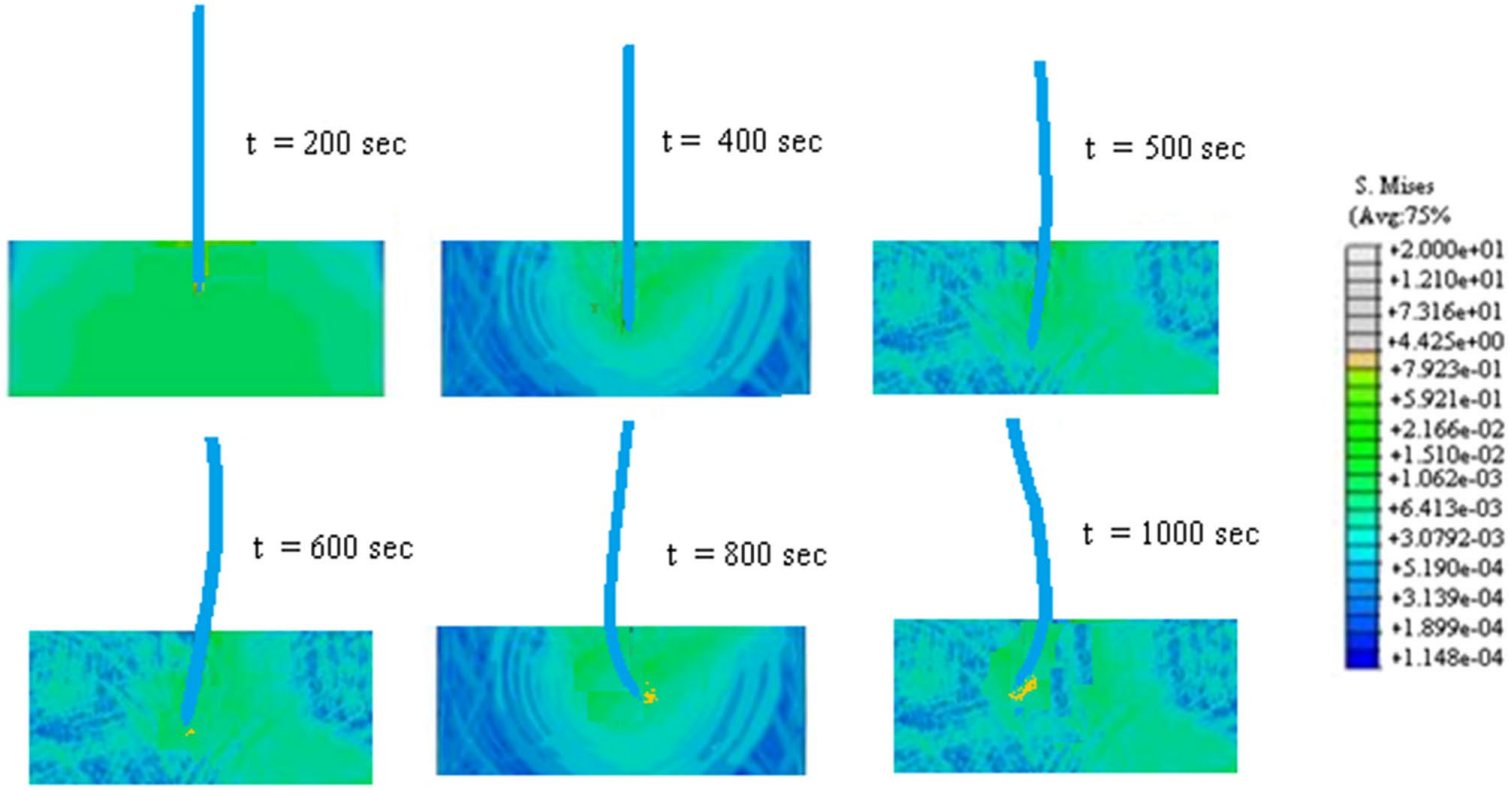
The deformation of the needle at $t =$ 400, 600, 800 and 1000 s.

The deformation of the needle at $t =$ 400, 600, 800 and 1000 s. are represented in Fig. 13.

## Conclusions

As mentioned in the introduction, the purpose of the present study is to investigate the navigation of a flexible needle into the human liver. In our analysis, we adopted the Cosserat elasticity to describe the interaction between the needle and the human liver. The theory incorporates the local rotation of points and the couple stress as well as the force stress representing the chiral properties of the human liver. However, since the liver is a deformable body that needs to be mechanically characterized, its elastic properties and deformation are evaluated. Estimates of the elastic constants have been made for a healthy liver in the absence of tumors. Computations result from the pseudopotential energy by retaining only the predominant terms in the evaluation of the elastic constants.
